# A Novel INCNS Score for Prediction of Mortality and Functional Outcome of Comatose Patients

**DOI:** 10.3389/fneur.2020.585818

**Published:** 2021-01-15

**Authors:** Zhihan Zhao, Xiao Zhang, Changgeng Song, Jingjing Zhao, Qiong Gao, Wen Jiang

**Affiliations:** Department of Neurology, Xijing Hospital, The Forth Military Medical University, Xi'an, China

**Keywords:** APACHE II, INCNS, GCS, FOUR, coma, outcome prediction, mortality

## Abstract

**Objectives:** The purpose of this study was to verify the veracity and reliability of the INCNS score for prediction of neurological ICU (NICU) mortality and 3-month functional outcome and mortality in comatose patients.

**Methods:** In this prospective study, data of the patients admitted to NICU from January 2013 to January 2019 were collected for validation. The 3-month functional outcomes were evaluated using modified Rankin Scale (mRS). By using the receiver operating characteristics curve (ROC) analysis, we compared the INCNS score with Glasgow Coma Scale (GCS), Full Outline of Un-Responsiveness Score (FOUR) and Acute Physiology and Chronic Health Evaluation II (APACHE II) for assessment of the predictive performance of these scales for 3-month functional outcome and mortality and NICU mortality performed at 24- and 72-h after admission to the NICU.

**Results:** Totally 271 patients were used for evaluation; the INCNS score achieved an AUC (area under the receiver operating characteristic curve) of 0.766 (95% CI: 0.711–0.815) and 0.824 (95% CI: 0.774–0.868) for unfavorable functional outcomes, an AUC of 0.848 (95% CI: 0.800–0.889) and 0.892 (95% CI: 0.848–0.926) for NICU mortality, and an AUC of 0.811 (95% CI: 0.760–0.856) and 0.832 (95% CI: 0.782–0.874) for the 3-month mortality after discharge from the NICU at 24- and 72-h. The INCNS score exhibited a significantly better predictive performance of mortality and 3-month functional outcomes than FOUR and GCS. There was no significant difference in predicting NICU mortality and 3-month functional outcomes between INCNS and APACHE II, but INCNS had better predictive performance of 3-month mortality than APACHE II.

**Conclusions:** The INCNS score could be used for predicting the functional outcomes and mortality rate of comatose patients.

## Introduction

Coma is a state of deep and prolonged unconsciousness in which a person cannot be awakened, unable to normally respond to sound, light or painful stimuli and comatose patients do not initiate actions voluntarily and lack normal sleep-wake cycle (1, 2). Since coma is not a condition that could recover quickly, it is of utmost importance to evaluate the prognosis and mortality of comatose patients so that subsequent treatments or other arrangements could be timely decided and executed (3). Currently, several strategies have been harnessed to predict the outcomes of comatose patients, such as electroencephalography (EEG), positron emission tomography (PET), and functional magnetic resonance imaging (fMRI) (4), but they require significant technological and computational expertise. In contrast, bedside behavioral evaluation is low cost, easy-to-implement, and remains the most commonly used method for consciousness assessment (5, 6).

The Glasgow Coma Scale (GCS) is major prognostic marker for comatose patients (7). However, its reliability and correlation with patient outcomes has been challenged lately because of its lack of information about potentially important clinical indicators such as brainstem reflexes, breathing patterns, and need of mechanical ventilation (8, 9). The Full Outline of Un-Responsiveness Score (FOUR) has been proven to be more reliable in assessing neurocritically ill patients than GCS mainly because of its inclusion of neurological reflex and respiratory examinations (6, 10). Although FOUR contains neurological functional scores, it still lacks the assessment of systemic conditions. The Acute Physiology and Chronic Health Evaluation II (APACHE II) scoring system is one of the most commonly used scores for critically ill patients and contains systemic condition scoring that is missing in FOUR, but it uses GCS as neurological functional scoring item, which lacks brainstem reflex examination. This might affect the prognostic accuracy of APACHE II (11).

In March 2019, our team developed the Inflammation, Nutrition, Consciousness, Neurological function, and Systemic function (INCNS) scale for evaluation of the outcomes of NICU patients. The INCNS was previously reported to have significantly greater prediction power than APACHE II at both 24- and 72-h in neurocritically ill patients (12). However, it is yet to be determined whether the INCNS could be used to predict the outcomes of comatose patients. In this study, we compared the INCNS score with the GCS, FOUR, and APACHE II scores of comatose patients at 24- and 72-h after admission to the NICU and assessed the prognostic performance of INCNS scores in predicting the functional outcome and mortality of comatose patients.

## Methods

### Patients

This study was based on a prospective database of consecutive comatose patients admitted to the NICU of Xijing Hospital, a tertiary-care center in Xian, China from January 2013 to January 2019. Patients who met the following diagnostic criteria of coma were included: based on the different portions of the GCS, namely: eye response value = 1, verbal response value ≤ 2, and motor response value ≤ 4 (3). Patients who stayed in the NICU for <72-h or patients with drug induced coma were excluded from the study.

The study protocol was reviewed and approved by the ethics committee of Xijing Hospital. All the procedures were executed according to Chinese laws and the Helsinki Declaration relative to patients' rights. Patient data were anonymized in the paper.

### Data Collection

The consciousness of patients was assessed on arrival to the emergency room (ER) by our neurocritical consultant with 3 or more years of experience. Blood tests were conducted and patient baseline data were collected upon NICU admission. The INCNS, APACHE II, FOUR, and GCS of each patient were evaluated by the same NICU neurologist who had received prior training in using INCNS, APACHE II, FOUR, and GCS for patient evaluation. We recorded the worst scores of each patient in the initial 24- and 72-h in the NICU which was used in the validation analysis. The INCNS scoring system is comprised of five domains: inflammation, nutrition, consciousness, neurological function, and systemic function. It consists of 19 items and has a maximum score of 44 (Table 1). The scoring system was originally designed for the prediction of the 3-month functional outcome of neurocritically ill patients (12).

**Table 1:** INCNS score sheet.

**INCNS score**					
	**Variables**	**Points**			
		**0**	**1**	**2**	**3**
Inflammation	WBC (10^9^/L)	4–10	2.9–3.9, 10.1–25.0	≤ 35.9, 38.5–40	–
	Temperature (axillary, °C)	36–38.4	≤35.9, 38.5–40	≥40.1	–
Nutrition	Albumin (g/L)	≥35	25–34.9	≤24.9	–
Consciousness	Arousal	Spontaneous eye opening	Eye opening to verbal command	Eye opening to pain	None
	Awareness	Correct response to question or command^a^	confused response to question or command^a^	Non-reflex Movements^b^	None
Neurologic function	Pupillary light reflex	Bilateral responsive	–	Unilateral slow/absent	Bilateral slow /absent
	Corneal reflex	Bilateral responsive	–	Unilateral slow/ absent	Bilateral slow /absent
	Verbal response	Accurate speech	Confused/inappropriate speech	Incomprehensible speech/none	–
	Motor response^c^	Unilateral/bilateral muscle strength scores ≥ 4	Unilateral/bilateral muscle strength scores of 2–3	Unilateral muscle strength scores ≤ 1	Bilateral muscle strength scores ≤ 1
		Obeying to command	Localizing to/withdrawal from pain	Flexing/extending to pain	None
	Swallowing function^d^	Water swallow test I-II	Water swallow test III-IV/unable to assess	–	–
	Respiration	Not intubated, 12–24	Not intubated, ≤ 11/≥25	Breathes above ventilator rate	Breathes at ventilator rate/apnea
Systemic condition	Age (y)	≤44	45–64	65–74	≥75
	Heart rate	60–100	40–59, 101–149	≤39, ≥150	–
	SBP (mm Hg)	90–140	70–89, 141–199	≤69, ≥200	–
	Blood glucose (mmol/L)	3.9–11.1	2.2–3.8, 11.2–19.3	≤2.1, ≥19.4	–
	Serum sodium (mmol/L)	130–150	120–129, 151–159	≤119, ≥160	–
	Serum potassium (mmol/L)	3.5–5.5	2.5–3.4, 5.6–6.9	≤2.4, ≥7.0	–
	Serum creatinine (μmol/L)	4.4–132	≤43, 133–171	≥172	–
	Total bilirubin (μmol/L)	≤34.1	34.2–102.5	≥102.6	–

*SBP, systolic blood pressure; WBC, white blood cell*.

a *The examiner may the patient their name of command the patient to move eyeballs and/or hands, if appropriate*.

b *Include evidence of visual pursuit or non-contingent behaviors*.

c *Muscle strength test is based on Lovett's scale (13). Either the muscle strength test or motor response to painful stimulus is performed on each patient*.

d *Swallowing function test is based on Water Swallow Test from Kubota (14)*.

The treatment procedures of comatose patients were carried out according to corresponding etiology (15).

### Outcomes Assessment

The 3-month neurological outcome was evaluated using the modified Rankin Scale (mRS) after the patients were discharged from the NICU by a trained physician who were blinded to patient clinical data. A mRS score <3 indicated a favorable outcome while a mRS score >2 indicated an unfavorable outcome (12). NICU mortality was defined as death before discharge from the NICU (16).

### Statistical Analysis

Descriptive data were shown as median (interquartile range, IQR) or mean ± standard deviation (SD) for continuous variable. Non-normally distributed variables were analyzed using Mann-Whitney U test; normally distributed variables were analyzed using Student *t-*test. Categorical variables were shown as percentages, and were analyzed and recorded using χ^2^-test, and Fisher's exact tests, where appropriate. All tests were two-sided, and a *P*-value of < 0.05 was considered statistically significant.

The receiver operating characteristics curve (ROC) analysis was used on the APACHE II, FOUR, GCS, and INCNS scores to determine their predictive power. We expanded the analysis of INCNS and APACHE II by calculating the sensitivity (Se), specificity (Sp), positive predictive value (PPV), negative predictive value (NPV), the number of correctly classified (CC) patients, and the maximum accuracy determined by cut-off values (Youden Index). We compared sensitivity, specificity, and CC using Mc Nemar's test (17), and examine the significance of difference for PPV and NPV using a modification of Wald tests (18). A *P*-value of < 0.0083 was considered statistically significant for ROC analysis, and a *P*-value of < 0.05 was considered statistically significant for the examination of Se, Sp, CC, PPV, and NPV. Statistical analyses were carried out using SPSS 17.0 and Medcalc 15.

## Results

### Demographic and Baseline Information

Two hundred seventy-six comatose patients stayed at least 72-h at our NICU from January 2013 to January 2019. Five patients were lost to follow up and excluded. Finally, 271 patients were included in the analysis. The median age for the cohort was 56 (IQR 40–70) years, and 160 (59%) were male. The median duration of hospital stay was 15 (IQR 7–28) days, and the median duration of NICU stay was 13 (IQR 7–25) days. Three months after NICU discharge, 225 (83%) patients had unfavorable outcome, and 122 (45%) patients died, including 26 (9.6%) deaths in the NICU.

The main causes of coma were cerebral infarction (32.5%), followed by central nervous system (CNS) infection (23.2%), and cerebral hemorrhage (18%). The main causes of in-hospital death were cerebral infarction (53.8%), followed by cerebral hemorrhage (19.2%). Although the incidence of CNS infection was higher than that of cerebral hemorrhage, the mortality of patients with CNS infection (15.4%) in the NICU was lower than that of patients with cerebral hemorrhage. In terms of 3-month functional outcomes and mortality, the results were similar to those of in-hospital mortality. Cerebral infarction was still the main cause of poor prognosis (37.8%) and mortality (45.9%), followed by cerebral hemorrhage (poor prognosis: 19.6%; mortality: 16.4%) and CNS infection (poor prognosis: 18.7%; mortality: 13.9%). The outcome of CNS infection was more favorable than that of cerebral hemorrhage after appropriate treatment.

Patient baseline characteristics and the worst values of all variables concerning the INCNS score during the initial 24- and 72-h in the NICU are shown in Supplementary Tables 1, 2, respectively. The etiology distribution of this cohort is presented in Supplementary Table 3.

### The Prognostic Performance of Functional Outcome for INCNS, APACHE II, FOUR, and GCS

**Figure 1:**
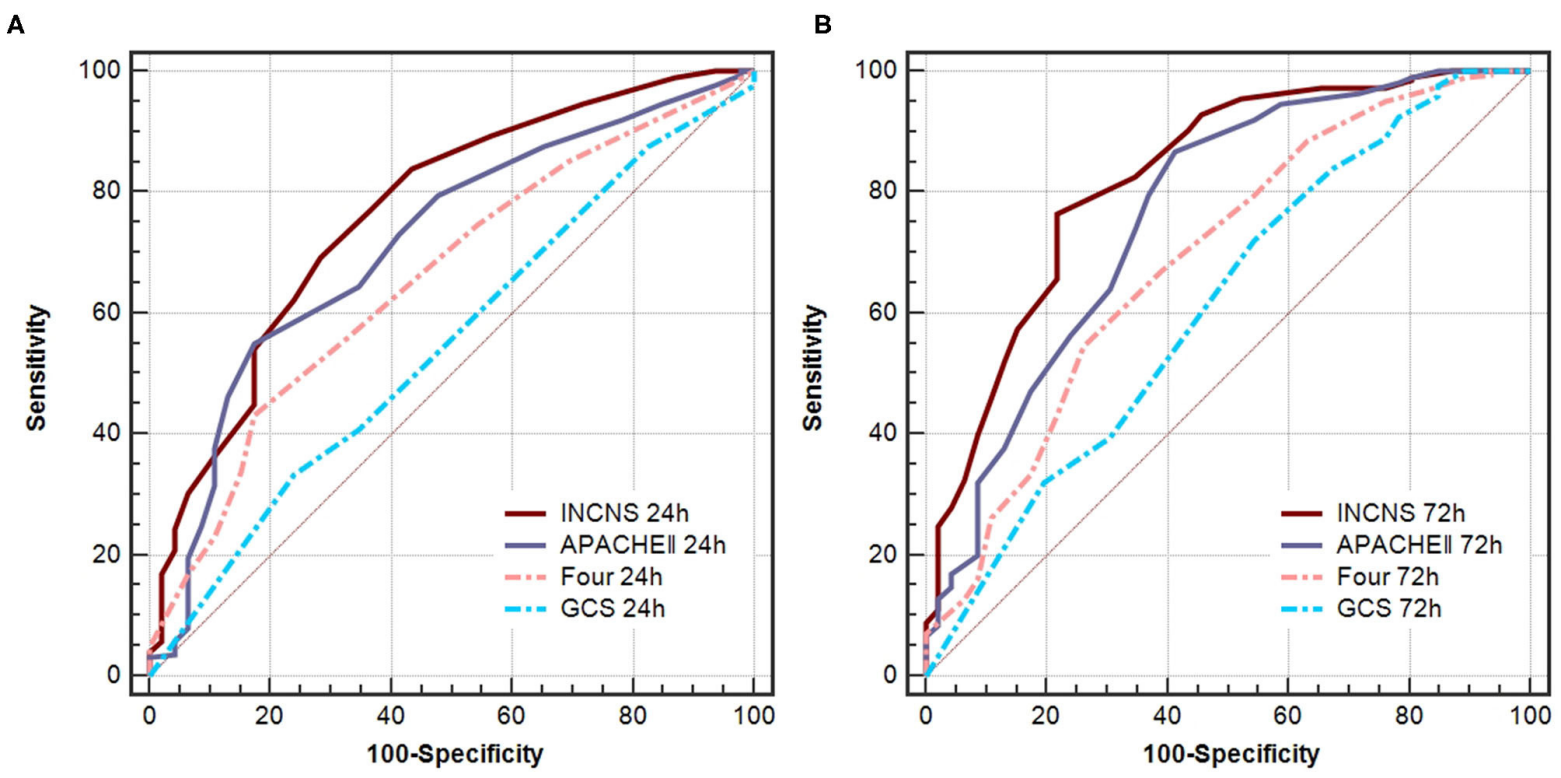
Comparisons of AUC for INCNS, APACHE II, FOUR, and GCS to distinguish the 3-month functional outcome in comatose patients. **(A)** 24-h ROC: *P*-value for the INCNS score AUC compared with APACHE II, FOUR, GCS is 0.1419, 0.0004, and <0.0001, respectively. **(B)** 72-h ROC: *P*-value for the INCNS score AUC compared with APACHE II, FOUR, GCS is 0.0496, <0.0001, and <0.0001, respectively. Level of significance corrected for multiple testing *p* < 0.0083.

ROC curves were used to predict the 3-month unfavorable functional outcomes of these four scores (Figure 1). The INCNS score yielded an area under the ROC curve (AUC) of 0.766 (0.711–0.815) for the initial 24-h NICU stay and an AUC of 0.824 (0.774–0.868) for the 72-h NICU stay. The 24- and 72-h APACHE II score yielded an AUC of 0.715 (0.657–0.768) and 0.764 (0.709–0.813), respectively. The 24- and 72-h FOUR score yielded an AUC of 0.658 (0.598–0.714) and 0.690 (0.631–0.745), resepctively. The 24- and 72-h GCS score yielded an AUC of 0.549 (0.487–0.609) and 0.613 (0.553–0.672).

According to the above results, the 72-h INCNS had the greatest prognostic performance for functional outcome, followed by the 24-h INCNS and the 72-h APACHE II. The INCNS score showed a much better discriminative performance compared with GCS (24-h: *P* < 0.001; 72-h: *P* < 0.001) and FOUR (24-h: *P* < 0.001; 72-h: *P* < 0.001). However, INCNS was not significantly better than APACHE II in functional outcome prediction at both 24-h (*P* = 0.142) and 72-h (*P* = 0.05) (Figure 1).

### The Prediction of NICU Mortality for INCNS, APACHE II, FOUR, and GCS

**Figure 2:**
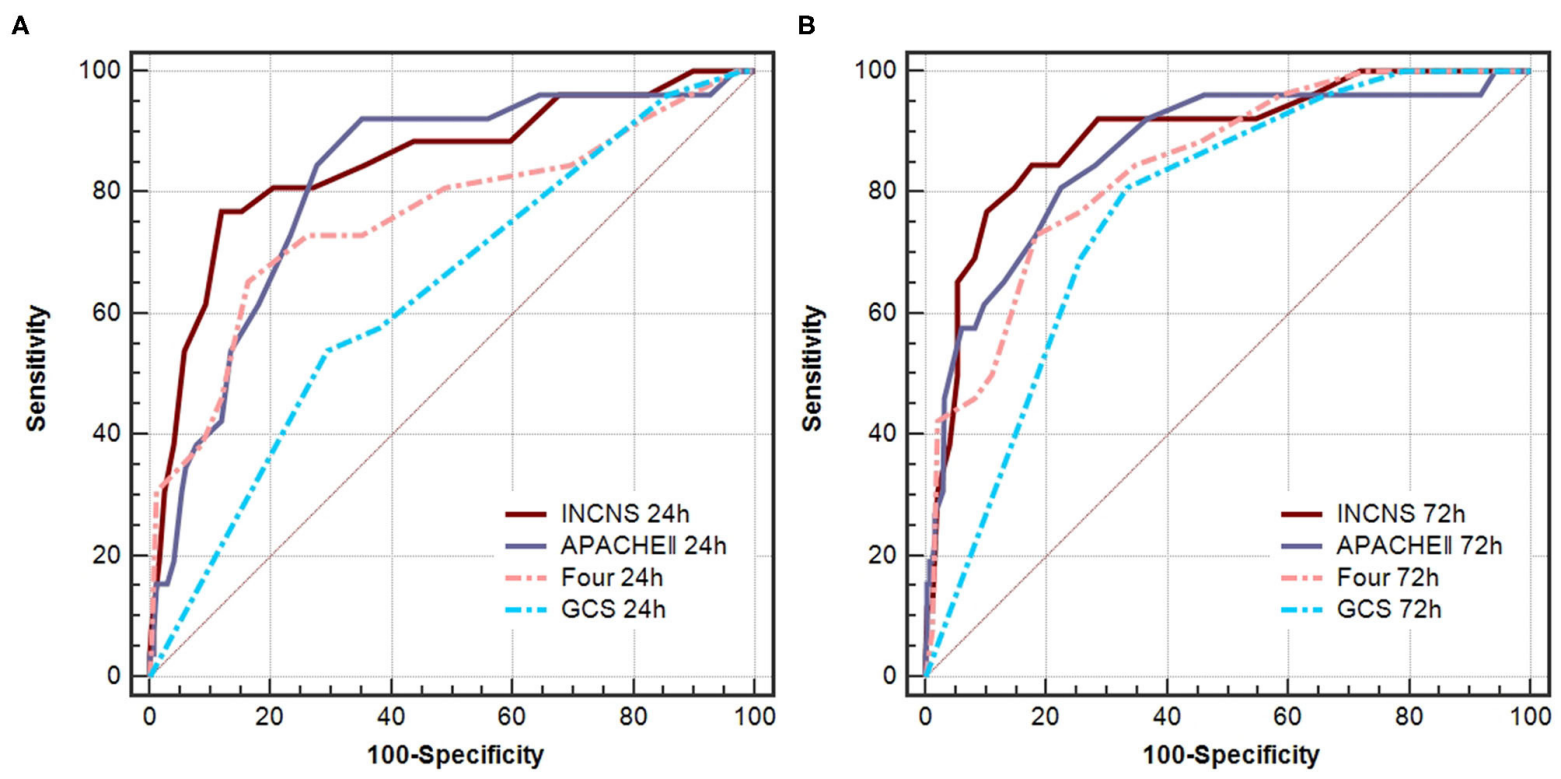
Comparisons of AUC for INCNS, APACHE II, FOUR and GCS to distinguish NICU mortality in comatose patients. **(A)** 24-h ROC: *P*-value for the INCNS score AUC compared with APACHE II, FOUR, GCS is 0.4257, 0.0033, and 0.0001, respectively. **(B)** 72-h ROC: *P*-value for the INCNS score AUC compared with APACHE II, FOUR, GCS is 0.3779, 0.0115, and 0.0009, respectively. Level of significance corrected for multiple testing *p* < 0.0083.

ROC curves were used to predict NICU mortality of these four scores (Figure 2). The INCNS score yielded an AUC of 0.848 (0.800–0.889) for the initial 24-h NICU stay and 0.892 (0.848–0.926) the 72-h NICU stay. The 24- and 72-h APACHE II score yielded an AUC of 0.820 (0.769–0.864) and 0.865 (0.819–0.903), respectively. The 24- and 72-h FOUR score yielded an AUC of 0.761 (0.705–0.810) and 0.842 (0.793–0.883), respectively. The 24- and 72-h GCS score yielded an AUC of 0.635 (0.574–0.692) and 0.772 (0.718–0.821), respectively (Figure 2). There was no significant difference between the INCNS score and APACHE II (24-h: *P* = 0.426, 72-h: *P* = 0.378) for predicting NICU mortality. However, the INCNS score showed a better discriminative performance compared with GCS (24-h: *P* < 0.001; 72-h: *P* < 0.001) and FOUR score (24-h: *P* = 0.003; 72-h: *P* = 0.012) (Figure 2).

### The Prediction of 3-month Mortality After Discharge From the NICU for INCNS, APACHE II, FOUR, and GCS

**Figure 3:**
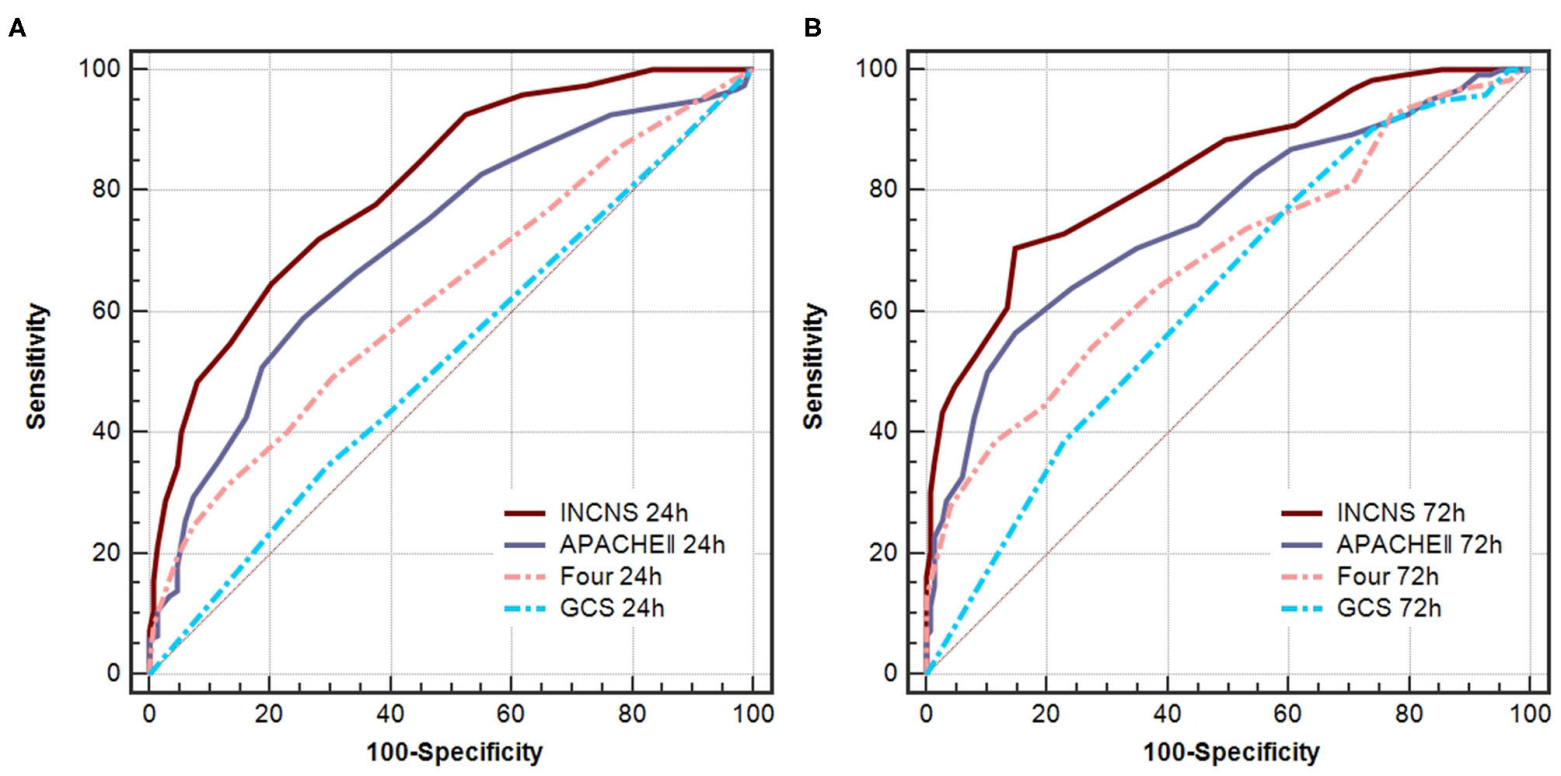
Comparisons of AUC for INCNS, APACHE II, FOUR and GCS to distinguish mortality after 3-months discharge from the NICU in comatose patients. **(A)** 24-h ROC: *P*-value for the INCNS score AUC compared with APACHE II, FOUR, GCS is 0.0001, <0.0001, and <0.0001, respectively. **(B)** 72-h ROC: *P*-value for the INCNS score AUC compared with APACHE II, FOUR, GCS is 0.0012, <0.0001, and <0.0001, respectively. Level of significance corrected for multiple testing *p* < 0.0083.

ROC curves were used to predict the 3-month mortality after discharge from the NICU of these four scores (Figure 2). The INCNS score yielded an AUC of 0.811 (0.760–0.856) for the initial 24-h NICU stay and 0.832 (0.782–0.874) for the 72-h NICU stay. The 24- and 72-h APACHE II score yielded an AUC of 0.713 (0.655–0.766) and 0.754 (0.698–0.804), respectively. The 24- and 72-h FOUR score yielded an AUC of 0.626 (0.565–0.684) and 0.684 (0.625–0.739), respectively. The 24- and 72-h GCS score yielded an AUC of 0.521 (0.459–0.581) and 0.625 (0.564–0.682), respectively (Figure 3). The INCNS score had greater predictive power than APACHE II, FOUR, and GCS scores at both 24- and 72-h (APACHE II 24-h: *P* < 0.001, 72-h: P = 0.001; FOUR 24-h: *P* < 0.001, 72-h: *P* < 0.001; GCS 24-h: *P* < 0.001, 72-h: *P* < 0.001) for predicting the 3-month mortality after discharge from the NICU (Figure 3).

### Further Comparison of INCNS and APACHE II

Based on the results of ROC analyses, the INCNS had the greatest discriminative power in both functional outcomes and mortality tests. The overall performances of INCNS, APACHEII, FOUR, and GCS are presented in Figures 1–3. The GCS and FOUR scores displayed a poorer performance than the INCNS and APACHE II scores. Therefore, further tests did not include GCS and FOUR scores.

**Figure 4:**
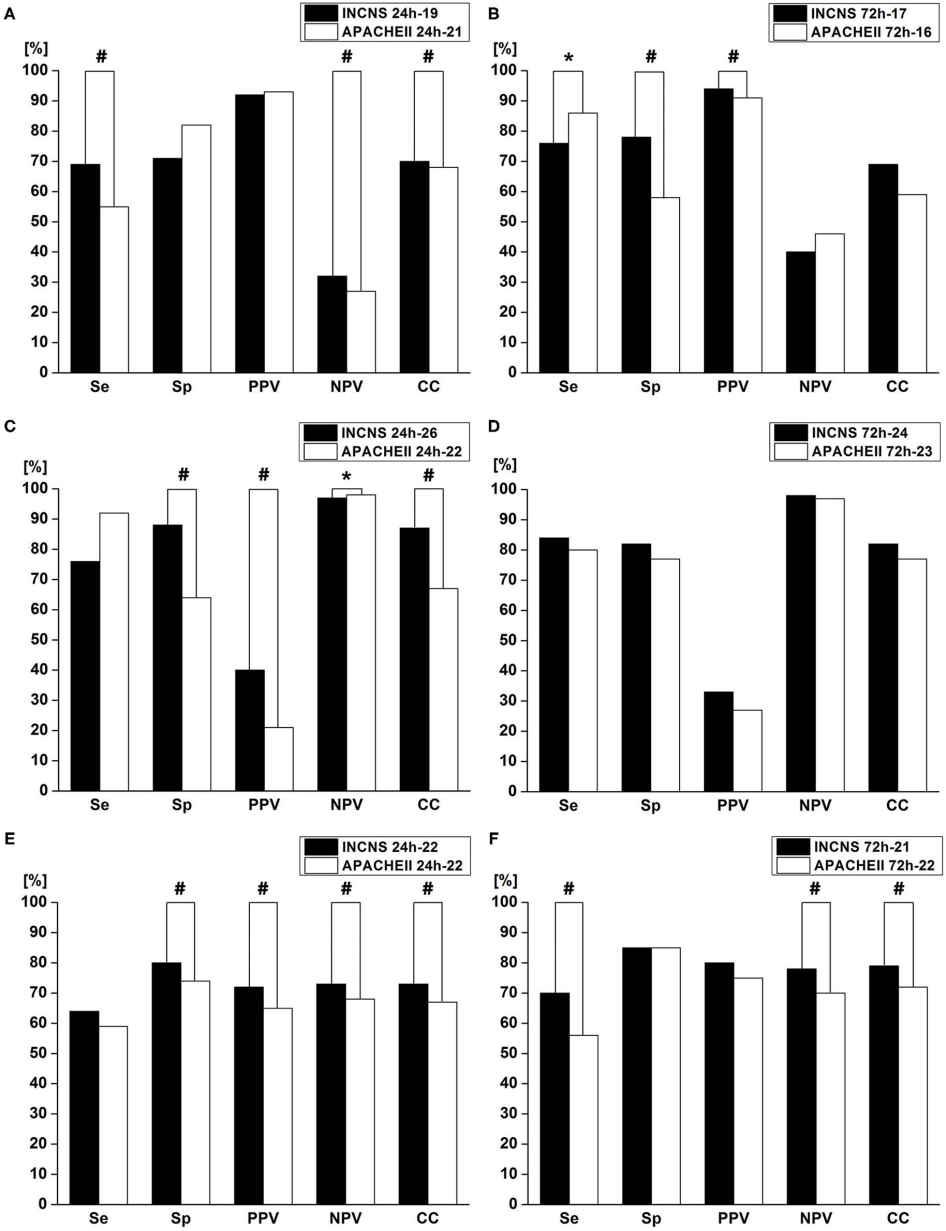
Comparisons of sensitivity(Se), specificity(Sp), positive predictive value (PPV), negative predictive value (NPV), and number of correctly classified (CC) patients between INCNS, and Acute Physiology and Chronic Health Evaluation II (APACHE II) to identify the predictive performance of INCNS; **(A,B)** 3-month functional outcome in comatose patients; **(C,D)** NICU mortality in comatose patients; **(E,F)** 3-month mortality in comatose patients. Twenty-four hours Functional outcome: Se: ^#^*p* < 0.0001, NPV: ^#^*p* = 0.0011, CC: ^#^*p* = 0.0015; 72 h Functional outcome: Se: *p = 0.0003, Sp: ^#^*p* < 0.0001, PPV: ^#^*p* < 0.0001; 24 h NICU mortality: Sp: ^#^*p* < 0.0001, PPV: ^#^*p* < 0.0001, NPV: *p = 0.0003, CC: ^#^*p* < 0.0001; 24 h 3-month mortality: Sp: ^#^*p* = 0.0039, PPV: ^#^*p* = 0.0009, NPV: ^#^*p* = <0.0001, CC: ^#^*p* = 0.0014; 72 h 3-month mortality: Se: ^#^*p* = 0.0029, NPV: ^#^*p* = <0.0001, CC: ^#^*p* = 0.0243. *P*-value < 0.05 was considered statistically significant.

The maximum sum of Se and Sp determines the cut-off value. The cut-off value of the 24- and 72-h INCNS for functional outcome was 19 and 17, respectively; the cut-off value of the 24- and 72-h INCNS for the 3-month mortality was 22, and 21, respectively; the cut-off value of the 24- and 72-h INCNS for NICU mortality was 26 and 24, respectively. Based on the cut-off value, the 24-h INCNS score had a predictive accuracy of 70.5% (Se: 69.3%, Sp: 71.7%, PPV: 92.3%, NPV: 32.4%, CC: 69.7%) for functional outcome and 82.6% (Se: 76.9%, Sp: 88.2%, PPV: 40.8%, NPV: 97.3%, CC: 87.1%) for NICU mortality; the 72-h INCNS score had a predictive accuracy of 77.4% (Se: 76.4%, Sp: 78.3%, PPV: 94.5%, NPV: 40.4%, CC: 76.8%) for functional outcome and 83.6% (Se: 84.6%, Sp: 82.5%, PPV: 33.8%, NPV: 98.1%, CC: 82.7%) for NICU mortality. For the 3-month mortality, the 24- and 72-h INCNS score had a predictive accuracy of 72.4% (Se: 64.8%, Sp: 79.9%, PPV: 72.5%, NPV: 73.5%, CC: 73.1%) and 77.9% (Se:70.5%, Sp: 85.2%, PPV: 79.6%, NPV: 77.9%, CC: 78.6%), respectively. APACHE II had poor performance when compared with INCNS, except in the comparison of 72-h Se (*P* = 0.003) for functional outcome, and 24-h NPV (*P* < 0.0001) for NICU mortality, which were shown to favor APACHE II (Figure 4 and Supplementary Table 4). Overall, INCNS had a better predictive performance than APACHE II.

## Discussion

In this study, we tested the INCNS score for its prognostic reliability of the 3-month functional outcome and mortality as well as NICU mortality rate in comatose patients, and then compared the INCNS score with GCS, FOUR, and APACHE II scores based on their prognostic value concerning comatose patients. Data from 271 comatose patients showed that the INCNS score had a significantly higher predictive ability for functional outcome and mortality rate than GCS and FOUR scores at both 24- and 72-h. The INCNS scoring system is more outstanding than APACHE II to predict 3-month mortality, but both scales performed equally well in evaluation of NICU mortality rate and functional outcome. In the analysis of predictive accuracy by cut-off values, INCNS was much better than APACHE II. Taken together, these results suggest that the INCNS scores could be a reliable tool to assess the outcome of comatose patients.

GCS was first used to evaluate patients with brain injury, and then widely used to evaluate consciousness disorder of different etiologies (19). The GCS has been proven to be a good outcome predictor for patients with coma (7, 19), but over the years, the deficiencies of GCS has also come to focus because it lacks many important clinical indicators (e.g., brainstem reflexes, eye movements, and breathing patterns). A more detailed scale, the FOUR score, was developed to be a supplement to GCS (8). Several studies have validated FOUR as a reliable score in predicting the outcome of comatose patients (7, 20–22). Previous report has also shown that FOUR and GCS performed equally well in predicting the outcome of comatose patients (7, 23). The APACHE II score is a clinical scoring system that is widely used in the ICU to reflect the severity of disease in patients (11). It is also used to predict the outcome of comatose patients. Michael et al. reported that APACHE II was associated with 3-month mortality in patients with severe traumatic brain injury, but GCS was not (24). A recent study has also shown that APACHE II can independently predict 1-month recovery of consciousness in patients with acute coma, while GCS and FOUR cannot (25). These results reflect that systemic condition is a very important indicator for predicting outcome in critically ill patients. In our study, APACHE II performed much better than GCS, and slightly better than FOUR in predicting the outcome of comatose patients (data not shown). This is consistent with previous report (25). Our study also showed that the AUC of GCS and FOUR scores was quite low when compared with previous reports; the FOUR score had greater discriminative power than GCS in predicting the outcome of comatose patients, which is inconsistent with previous results (7, 21). Our NICU receives many patients that cannot be handled by other NICUs in our area and neighboring provinces, so our patients generally have very low neurological function score and severe systemic complications. The FOUR score is proved to perform better than GCS at very low scores because GCS lacks brainstem reflexes, which is an important component in the FOUR score (7). Systemic condition evaluation is not included in GCS and FOUR; therefore, severe systemic complications may affect predictive capability of GCS and FOUR. So, this might explain why our results are inconsistent with other reports.

There are several scores for predicting the outcome of patients in the ICU, such as APACHE II and Simplified Acute Physiology Score II (SAPS II) (26). But the lack of assessment of neurological deficits in those scores may bias the outcome prediction in patients with neurocritical illnesses. In 2019, we developed the INCNS score for prediction of 3-month functional outcome in neurocritically ill patients. It contains 19 items, which covers inflammation, nutrition, consciousness, neurological function, and systemic function (12). In our previous study, the INCNS score had a significantly stronger predictive power than FOUR, GCS, and APACHEII in patients with neurocritical illnesses, which might be due to the fact that the INCNS scoring system incorporates both respiratory and neurological reflex functions. Because dysphagia has a great impact on the prognosis of patients, water swallow test was also included. Aside from this, we added systemic function scores, which were proven to be of importance for predicting the outcome of critically ill patients from the APACHE II score. INCNS also included biomarkers which are associated with functional outcomes of common neurocritical illnesses (such as blood glucose and serum albumin) which APACHE II lacks (27, 28). The INCNS records the worst motor response from any limb while GCS, FOUR and APACHE II scores records the best. Because disability is an important factor for the prognosis, so it might also be one of the explanations for why the INCNS score showed a higher ability in predicting outcomes than the other three scores.

The prognosis of comatose patients is quite different. Some patients gradually come out of the coma, some progress to the vegetative state, and others die (1, 2). Therefore, it is very difficult to judge the prognosis of comatose patients, especially patients with neurocritical illnesses. Although the INCNS score was shown to be useful in predicting functional outcomes in patients with neurocritical illnesses in our previous study, these patients have different states of consciousness: some patients are in coma, some in the vegetative state, and others are awake. The previous study was not focused on comatose patients. The ability of the INCNS score to predict 3-month functional outcomes of comatose patients was not clear. The predictive power of INCNS for mortality was not tested either (12). In order to expand the application of the score, we compared the predictive capability of INCNS with GCS, FOUR, and APACHE II scores in patients with coma. As expected, the INCNS performed much better than GCS and FOUR scores. In the analysis of predictive accuracy by cut-off values, INCNS performed better than APACHE II. INCNS also has a much greater predictive power than APACHE II for predicting the 3-month mortality after discharge from the NICU. However, INCNS was not better than APACHE II for predicting 3-month functional outcomes in comatose patients. In the test of NICU mortality, INCNS did not perform better than the APACHE II either. The difference between the two scores is not as obvious as previously reported (12). Compared with APACHE II, some modifications of INCNS may not have clinical significance for comatose patients, such as water swallow test. But INCNS is more concise and easier to operate. APACHE II has a total score of 71, while INCNS has only a score of 44. INCNS changed the mean arterial pressure into systolic blood pressure and did not include blood gas analysis to avoid related calculation. So, in conclusion, the INCNS could be a useful tool to predict the outcome of comatose patients.

The APACHE II score was established based on the worst recordings during the first 24-h in general ICU (11). But some studies suggested that the prognostic score, such as the APACHE II score, performed at 72-h in admission to the ICU showed a much better accuracy rate than at 24- and 48-h in admission (29, 30). Our study also found that the INCNS, APACHE II, GCS, and FOUR scores at 72-h had a greater discriminative power vs. 24-h. The probable explanation might be that patients with neurocritical illnesses were likely to worsen beyond 24-h after disease onset because of primary neurological damage or complications. Therefore, it is more appropriate to perform the prognostic score at 72-h after admission instead of 24-h.

Although the INCNS scoring system demonstrated an overall better predictive value of prognosis and mortality than the GCS, FOUR, and APACHE II scores, it still has some shortcomings and limitations: (a) The predictor variables added in the INCNS scoring system has only been verified by our previous study and clinical experience (12). Further studies with larger sample size and longer follow-up duration are needed to validate the INCNS. (b) There are 19 items in the INCNS score; the excessive number of items may cause inconvenience for clinical practice. (c) Our study carry out in a single center at tertiary care institution. The study findings may not be generalizable to primary and secondary care settings, which would bias the conclusions of the regression model and hamper generalization. Further multicenter studies with a larger sample size are needed for the validation of the INCNS scoring system.

## Conclusion

The INCNS score had a higher predictive value for prognosis of comatose patients compared with the GCS, FOUR, and APACHE II scores. INCNS assessed at 72-h after admission to the NICU had better prognostic value than INCNS assessed at 24-h. The INCNS could serve as a practical prognostic tool to evaluate the functional recovery and mortality in patients with coma.

## Data Availability Statement

The raw data supporting the conclusions of this article will be made available by the authors, without undue reservation.

## Ethics Statement

The studies involving human participants were reviewed and approved by the ethics committee of Xijing hospital. Written informed consent to participate in this study was provided by the participants' legal guardian/next of kin. Written informed consent was obtained from the individual(s), and minor(s)' legal guardian/next of kin, for the publication of any potentially identifiable images or data included in this article.

## Author Contributions

WJ designed the study. ZZ and XZ collected patient's data and drafted the manuscript. QG ran data analyses. CS and JZ prepared the figures and revised the manuscript. All authors contributed to the article and approved the submitted version.

## Conflict of Interest

The authors declare that the research was conducted in the absence of any commercial or financial relationships that could be construed as a potential conflict of interest.
