# Supplementary material for: A Novel INCNS Score for Prediction of Mortality and Functional Outcome of Comatose Patients
Source: Front Neurol. 2021 Jan 15;11:585818. doi: 10.3389/fneur.2020.585818 (PMC7843913; doi:10.3389/fneur.2020.585818)
Supplement: Supplementary file 1 [file Data_Sheet_1.doc]

**SUPPLEMENTAL MATERIAL**

**Supplement tables**

Analyses of the enrolled comatose patients for verifying the INCNS score for prediction of their mortality and functional outcome

Table S1: Patient characteristics at 24h in NICU

| **NICU Mortality** | | | | |
| --- | --- | --- | --- | --- |
| **Variable** | **Total**  **(n=271)** | **Alive**  **(n=245)** | **Death**  **(n=26)** | ***p*** |
| Age, mean±SD, y | 56 (40,70) | 56 (39,70) | 57 (48,80) | 0.274 (Mann-Whitney U) |
| Gender, male, No. (%) | 160 (59) | 140 (57.1) | 20 (76.9) | 0.051 (χ2) |
| Hospital stay length, median (IQR),d | 15 (7, 28) | 15 (9, 29) | 5 (3, 10) | <0.001 (Mann-Whitney U) |
| NICU stay length, median (IQR),d | 13 (7, 25) | 14 (8, 28) | 4 (3, 9) | <0.001 (Mann-Whitney U) |
| Ventilated at day 1 yes/no | 72/199 | 58/187 | 14/12 | 0.001 (Mann-Whitney U) |
| GCS, median (IQR), points | 5 (3, 5) | 5 (3, 5) | 3 (3, 5) | 0.015 (Mann-Whitney U) |
| FOUR, median (IQR), points | 7 (5, 9) | 8 (5, 9) | 4 (1, 7) | <0.001 (Mann-Whitney U) |
| APACHE II, median (IQR), points | 20 (17, 24) | 20 (17, 23) | 26 (23, 30) | <0.001 (Mann-Whitney U) |
| INCNS, median (IQR), points | 20 (17, 24) | 20 (17, 23) | 28 (26, 30) | <0.001 (Mann-Whitney U) |
| **Prognosis after 3 months discharge from NICU** | | | | |
| **Variable** | **Total (n=271)** | **mRS:0-2 (n=46)** | **mRS:3-6 (n=225)** | ***p*** |
| Age, mean±SD, y | 56 (40,70) | 35 (22,57) | 59 (45,73) | <0.001 (Mann-Whitney U) |
| Gender, male, No. (%) | 160 (59) | 25 (54.3) | 135 (60) | 0.513 (χ2) |
| Hospital stay length, median (IQR),d | 15 (7, 28) | 16 (11,39) | 14 (7,27) | 0.047 (Mann-Whitney U) |
| NICU stay length, median (IQR), d | 13 (7, 25) | 14 (11,35) | 13 (7,24) | 0.044 (Mann-Whitney U) |
| Ventilated at day 1 yes/no | 72/199 | 7/39 | 65/160 | <0.001 (Mann-Whitney U) |
| GCS, median (IQR), points | 5 (3, 5) | 5 (4, 5) | 5 (3, 5) | 0.265 (Mann-Whitney U) |
| FOUR, median (IQR), points | 7 (5, 9) | 8 (7, 10) | 7 (5, 9) | 0.001 (Mann-Whitney U) |
| APACHE II, median (IQR), points | 20 (17, 24) | 17 (16, 20) | 21 (18, 25) | <0.001 (Mann-Whitney U) |
| INCNS, median (IQR), points | 20 (17, 24) | 16 (14, 19) | 21 (18, 24) | <0.001 (Mann-Whitney U) |
| **Mortality after 3 months discharge from NICU** | | | | |
| **Variable** | **Total**  **(n=271)** | **Alive**  **(n=149)** | **Death**  **(n=122)** | ***p*** |
| Age, median (IQR), y | 56 (40,70) | 50 (31,64) | 62 (51,76) | <0.001 (Mann-Whitney U) |
| Gender, male, No. (%) | 160（59） | 83（55.7） | 77（63.1） | 0.217 (χ2) |
| Hospital stay length, median (IQR),d | 15 (7, 28) | 19（11,31） | 9（4,22） | <0.001 (Mann-Whitney U) |
| N-ICU stay length, median (IQR),d | 13 (7, 25) | 17（11,29） | 8（4,18） | <0.001 (Mann-Whitney U) |
| Ventilated at day 1 yes/no | 72/199 | 33/116 | 39/83 | 0.002 (Mann-Whitney U) |
| GCS, median (IQR), points | 5 (3, 5) | 5（3,5） | 5（3,5） | 0.529 (Mann-Whitney U) |
| FOUR, median (IQR), points | 7 (5, 9) | 8（6,9） | 7（4,8） | <0.001 (Mann-Whitney U) |
| APACHE II, median (IQR), points | 20 (17, 24) | 19（17,22） | 23（20,27） | <0.001 (Mann-Whitney U) |
| INCNS, median (IQR), points | 20 (17, 24) | 18（15,21） | 23（20,27） | <0.001 (Mann-Whitney U) |

Abbreviations. APACHE II, Acute Physiology and Chronic Health Evaluation II; GCS, Glasgow Coma Scale; FOUR, Full Outline of UnResponsiveness; IQR, interquartile range; mRS, Modified Rankin Scale; NICU, neurological intensive care unit.

Table S2. Patient characteristics at 72h in NICU

| **NICU Mortality** | | | | |
| --- | --- | --- | --- | --- |
| **Variable** | **Total**  **(n=271)** | **Alive**  **(n=245)** | **Death**  **(n=26)** | ***p*** |
| GCS, median (IQR), points | 5 (3, 6) | 5 (3, 6) | 3 (3, 4) | <0.001(Mann-Whitney U) |
| FOUR, median (IQR), points | 8 (5, 9) | 8 (5, 10) | 4 (1, 5) | <0.001(Mann-Whitney U) |
| APACHE II, median (IQR), points | 20 (16, 23) | 19 (16, 22) | 28 (23, 31) | <0.001(Mann-Whitney U) |
| INCNS, median (IQR), points | 19 (15, 23) | 18 (15, 22) | 29 (26, 31) | <0.001(Mann-Whitney U) |
| **Prognosis** | | | | |
| **Variable** | **Total**  **(n=271)** | **mRS:0-2 (n=46)** | **mRS:3-6 (n=225)** | ***p*** |
| GCS, median (IQR), points | 5 (3, 6) | 5 (4, 7) | 5 (3, 6) | 0.013 (Mann-Whitney U) |
| FOUR, median (IQR), points | 8 (5, 9) | 9 (7, 11) | 7 (4, 9) | <0.001(Mann-Whitney U) |
| APACHE II, median (IQR), points | 20 (16, 23) | 15 (12, 19) | 20 (17, 24) | <0.001(Mann-Whitney U) |
| INCNS, median (IQR), points | 19 (15, 23) | 13 (11, 16) | 20 (17, 25) | <0.001(Mann-Whitney U) |
| **Mortality after 3 months discharge from NICU** | | | | |
| **Variable** | **Total**  **(n=271)** | **Alive**  **(n=149)** | **Death**  **(n=122)** | ***p*** |
| GCS, median (IQR), points | 5 (3, 6) | 5（4,7） | 5（3,5） | <0.001(Mann-Whitney U) |
| FOUR, median (IQR), points | 8 (5, 9) | 8（6,10） | 6（3,9） | <0.001(Mann-Whitney U) |
| APACHE II, median (IQR), points | 20 (16, 23) | 18（15,20） | 23（18,27） | <0.001(Mann-Whitney U) |
| INCNS, median (IQR), points | 19 (15, 23) | 16（13,19） | 23（19,27） | <0.001(Mann-Whitney U) |

Abbreviations. APACHE II, Acute Physiology and Chronic Health Evaluation II; GCS, Glasgow Coma Scale; FOUR, Full Outline of UnResponsiveness; IQR, interquartile range; mRS, Modified Rankin Scale; NICU, neurological intensive care unit.

Table S3. Etiology distribution

| **NICU Mortality** | | | |
| --- | --- | --- | --- |
| **Aetiology** | **Total 271**  **No. (%)** | **Alive:245**  **No. (%)** | **Death:26**  **No. (%)** |
| Cerebral infarction | 88 (32.5) | 74 (30.2) | 14 (53.8) |
| Central nervous system infection | 63 (23.2) | 59 (24.1) | 4 (15.4) |
| Hypoxic ischemic encephalopathy | 22 (8.1) | 21 (8.6) | 1 (3.8) |
| Intracerebral hemorrhage | 49 (18.1) | 44 (18.0) | 5 (19.2) |
| toxic encephalopathy | 13 (4.8) | 13 (5.3) | 0 |
| Autoimmune encephalitis | 10 (3.7) | 10 (4.1) | 0 |
| unexplained encephalopathy | 5 (1.8) | 5 (2.0) | 0 |
| Traumatic brain injury | 3 (1.1) | 3 (1.2) | 0 |
| Other neurologic diseases | 18 (6.6) | 16 (6.5) | 2 (7.7) |
| **Prognosis** | | | |
| **Aetiology** | **Total 271**  **No. (%)** | **mRS (0-2): 46**  **No. (%)** | **mRS (3-6): 225**  **No. (%)** |
| Cerebral infarction | 88 (32.5) | 3 (6.5) | 85 (37.8) |
| Central nervous system infection | 63 (23.2) | 21 (45.7) | 42 (18.7) |
| Hypoxic ischemic encephalopathy | 22 (8.1) | 0 | 22 (9.8) |
| Intracerebral hemorrhage | 49 (18.1) | 5 (10.9) | 44 (19.6) |
| toxic encephalopathy | 13 (4.8) | 4 (8.7) | 9 (4.0) |
| Autoimmune encephalitis | 10 (3.7) | 7 (15.2) | 3 (1.3) |
| unexplained encephalopathy | 5 (1.8) | 0 | 5 (2.2) |
| Traumatic brain injury | 3 (1.1) | 0 | 3 (1.3) |
| Other neurologic diseases | 18 (6.6) | 6 (13.0) | 12 (5.3) |
| **Mortality after 3 months discharge from NICU** | | | |
| **Aetiology** | **Total 271**  **No. (%)** | **Alive:149**  **No. (%)** | **Death:122**  **No. (%)** |
| Cerebral infarction | 88 (32.5) | 32(21.5) | 56(45.9) |
| Central nervous system infection | 63 (23.2) | 46 (30.9) | 17 (13.9) |
| Hypoxic ischemic encephalopathy | 22 (8.1) | 11(7.4) | 11(9.0) |
| Intracerebral hemorrhage | 49 (18.1) | 29(19.5) | 20(16.4) |
| toxic encephalopathy | 13 (4.8) | 8(5.4) | 5(4.1) |
| Autoimmune encephalitis | 10 (3.7) | 8 (5.4) | 2 (1.6) |
| unexplained encephalopathy | 5 (1.8) | 1(0.7) | 4(3.3) |
| Traumatic brain injury | 3 (1.1) | 2(1.3) | 1(0.8) |
| Other neurologic diseases | 18 (6.6) | 12(8.1) | 6(4.9) |

Table S4. Overall performances of the INCNS and APACHE II scoring systems

| **Score** | **AUC (95% CI)** | **Cut-off values** | **Se** | **Sp** | **PPV** | **NPV** | **CC** | **Accuracy** |
| --- | --- | --- | --- | --- | --- | --- | --- | --- |
| **mRS** | | | | | | | | |
| INCNS 24h | 0.766  (0.711 – 0.815) | 19 | 69.3 | 71.7 | 92.3 | 32.4 | 69.7 | 70.5 |
| 72h | 0.824  (0.774 – 0.868) | 17 | 76.4 | 78.3 | 94.5 | 40.4 | 76.8 | 77.4 |
| APACHE II 24h | 0.715  (0.657 – 0.768) | 21 | 55.1 | 82.6 | 93.9 | 27.3 | 59.8 | 68.9 |
| 72h | 0.764  (0.709 – 0.813) | 16 | 86.2 | 58.7 | 91.1 | 46.6 | 81.5 | 72.5 |
| **NICU Mortality** | | | | | | | | |
| INCNS 24h | 0.848  (0.800-0.889) | 26 | 76.9 | 88.2 | 40.8 | 97.3 | 87.1 | 82.6 |
| 72h | 0.892  (0.848-0.926) | 24 | 84.6 | 82.5 | 33.8 | 98.1 | 82.7 | 83.6 |
| APACHE II 24h | 0.820  (0.769-0.864) | 22 | 92.3 | 64.9 | 21.8 | 98.8 | 67.5 | 78.6 |
| 72h | 0.865  (0.819-0.903) | 23 | 80.8 | 77.6 | 27.6 | 97.4 | 77.9 | 79.2 |
| **Mortality after 3 months discharge from NICU** | | | | | | | | |
| INCNS 24h | 0.811  (0.760-0.856) | 22 | 64.8 | 79.9 | 72.5 | 73.5 | 73.1 | 72.4 |
| 72h | 0.832  (0.782-0.874) | 21 | 70.5 | 85.2 | 79.6 | 77.9 | 78.6 | 77.9 |
| APACHE II 24h | 0.713  (0.655-0.766) | 22 | 59 | 74.5 | 65.5 | 68.9 | 67.5 | 66.8 |
| 72h | 0.754  (0.698-0.804) | 22 | 55.7 | 85.2 | 75.6 | 70.2 | 72.0 | 70.5 |

Abbreviations. AUC, area under the curve; CC, correctly classified; NPV, negative predictive value; PPV, positive predictive value; Se, sensitivity; Sp, specificity.
